# Development of new prognostic model based on pretreatment βLRI and LLRI for stage IE/IIE upper aerodigestive tract ENKTL, nasal type

**DOI:** 10.18632/oncotarget.16720

**Published:** 2017-03-30

**Authors:** Wumin Dai, Bo Jia, Jianliang Yang, Shengyu Zhou, Peng Liu, Xiaohui He, Yan Qin, Lin Gui, Changgong Zhang, Xiaohong Han, Yan Sun, Yuankai Shi

**Affiliations:** ^1^ Department of Medical Oncology, Beijing Key Laboratory of Clinical Study on Anticancer Molecular Targeted Drugs, National Cancer Center/Cancer Hospital, Peking Union Medical College and Chinese Academy of Medical Sciences, Beijing, 100021, China

**Keywords:** beta2-microglobin to lymphocytes ratio index, lactate dehydrogenase to lymphocytes ratio index, systemic immune-inflammation, prognosis, extranodal natural killer/T cell lymphoma

## Abstract

To identify simple non-invasive prognostic factors for extranodal natural killer/T cell lymphoma (ENKTL), we have investigated the prognostic value of pretreatment β2-microglobin to lymphocytes ratio index (βLRI) or lactate dehydrogenase to lymphocytes ratio index (LLRI), by analyzing the retrospective data from 211 ENKTL patients. Receiver operating characteristic (ROC) curve analysis was performed to determine the cut-off value of pretreatment βLRI and LLRI. The univariate analysis indicated that Ann Arbor Stage (*p* = 0.008), Eastern Cooperative Oncology Group score (ECOG) (*p* = 0.009), International Prognostic Index (IPI) (*p* = 0.023), βLRI (*p* = 0.003), LLRI (*p* = 0.04), neutrophil-lymphocyte ratio index (*p* = 0.025) and monocyte/granulocyte to lymphocyte ratio (*p* = 0.030) were significantly associated with overall survival (OS) in ENKTL patients. However, multivariate analysis demonstrated that only Ann Arbor Stage (*p* = 0.028), βLRI (*p* < 0.001) and LLRI (*p* = 0.006) were only correlated independently with OS. Furthermore, βLRI and LLRI based new prognostic model showed improved discrimination for stage IE/IIE upper aerodigestive tract in ENKTL patients than IPI and Korean Prognostic Index. Overall, our study concluded that new βLRI-based prognosis model is useful to stratify ENKTL patients and higher βLRI and LLRI can act as independent prognostic predictor candidates in early stage ENKTL.

## INTRODUCTION

Extranodal natural killer/T cell lymphoma (ENKTL) is a highly aggressive non-Hodgkin lymphoma, distinguished from other subtypes on the basis of its unique characteristics, such as predominant involvement of the nasal cavity and nasopharynx, high prevalence in East Asia and South America, and relationship to Epstein-Barr virus infection. Based on published literature, the treatment outcomes of ENKTL are generally poor, and vary widely [1–3]. Five-year overall survival (OS) rates in large cohort studies range from 30–86%, with most studies demonstrating the 5-year OS of < 50%. Thus, investigation of optimal therapeutic targets and prognostic factors for ENKTL is still warranted.

Importantly, two major prognostic models have been utilized for NK/T-cell lymphoma: The International Prognostic Index (IPI) and the Korean Prognostic Index (KPI). IPI has not gained widespread acceptance for ENKTL prognosis, as 60% of the ENKTL patients are grouped into low IPI risk categories (score, 0–1). However, the KPI model appears to be more useful for predicting ENKTL prognosis [4–6], as stage III or IV patients are included in the KPI model. However, some patients in the low KPI risk group still have poor clinical outcomes [7], which indicate that the scoring systems based on both these models should be further modified.

A growing body of evidence has shown the involvement of inflammation in occurrence and development of cancer, including ENKTL [8–10]. The microenvironment surrounding the tumor encompasses both tumor and host-derived cytokines, inflammatory cytokines, and infiltrating immune cells. Among the different cell types involved in tumor responses, lymphocytes basically accelerate antitumor immune response, and their presence closely relates with higher cytotoxic treatment and a more favorable prognosis [11]. In recent years, higher levels of serum β2-microglobulin (β2-MG) and lactate dehydrogenase (LDH) prior to treatment have been shown to correlate with poor prognosis in patients with malignancy [12–16].

β2-MG constitutes the light chain subunit of major histocompatibility complex (MHC) class I antigens and is present on the surface of all nucleated cells. Similarly LDH has been shown to be an indirect marker of hypoxia and neo-angiogenesis, which stimulates the proliferation, metabolism, and metastasis of tumor cells [17]. It has been proposed that combining multiple inflammatory marker levels can incrementally improve the prognostic value of well-established inflammation-based scoring systems [18]. To the best of our knowledge, there have been no studies assessing the prognostic value of pretreatment β2-MG to lymphocytes ratio (βLRI) and LDH to lymphocytes ratio (LLRI) in predicting survival of ENKTL patients. Thus, in this study we sought to evaluate the prognostic value of βLRI and LLRI in ENKTL patients.

## RESULTS

### Baseline characteristics

Our study recruited 211 patients, including 65 with limited stage IE, 94 with paranasal extension stage IE and 52 with stage IIE. Among them, 151 patients were males, and 60 were females with a median age of 42 years (range 11–85 years). A total of 102 patients (48.3%) displayed B symptoms, and the majority (96.2%) showed Eastern Cooperative Oncology Group score (ECOG) of 0–1. LDH levels were elevated in 56 patients (26.5%). The majority of patients (92.4%, 72%) were grouped into low or low-intermediate risk categories according to IPI and KPI, respectively. The baseline characteristics of these patients are shown in Table 1.

**Table 1:** Clinical characteristics of ENKTL patients

Characteristic		Patients (*n* = 211)
	*N*	%
**Age, years Median (Range)**	42 (11–85)	
≤ 45	132	62.6
> 45	79	37.4
**Gender**		
Male	151	71.6
Female	60	28.4
**B symptoms**		
Yes	102	48.3
No	109	51.7
**Ann Arbor Stage**		
IE	159	75.4
Limited	65	
**Paranasal extension**	94	
IIE	52	24.6
Limited	0	
Paranasal extension	52	
**ECOG**		
0–1	203	96.2
> 1	8	3.8
**Paranasal extension**		
Yes	146	69.1
No	65	30.8
**Lymph Node Infiltration**		
Yes	45	21.3
No	166	78.7
**LDH Elevated**		
Yes	56	26.5
No	147	69.7
Missing	8	3.8
**IPI**		
0	134	63.5
1	61	28.9
2	13	6.2
3	3	1.4
**KPI**		
1	83	39.3
2	69	32.7
3	44	20.9
4	15	7.1
**Treatment modality**		
Chemoradiotherapy	114	54
Radiotherapy	97	46
**Recurrence**		
Yes	56	26.5
No	155	73.5
**Survival**		
Yes	174	82.5
No	37	17.5

ENKL = extranodal natural killer/T-cell lymphoma, ECOG = Eastern Cooperative Oncology Group, LDH = lactate dehydrogenase, IPI = International Prognostic Index, KPI = Korean Prognostic Index.

### Determination of cut-off values

**Figure 1:**
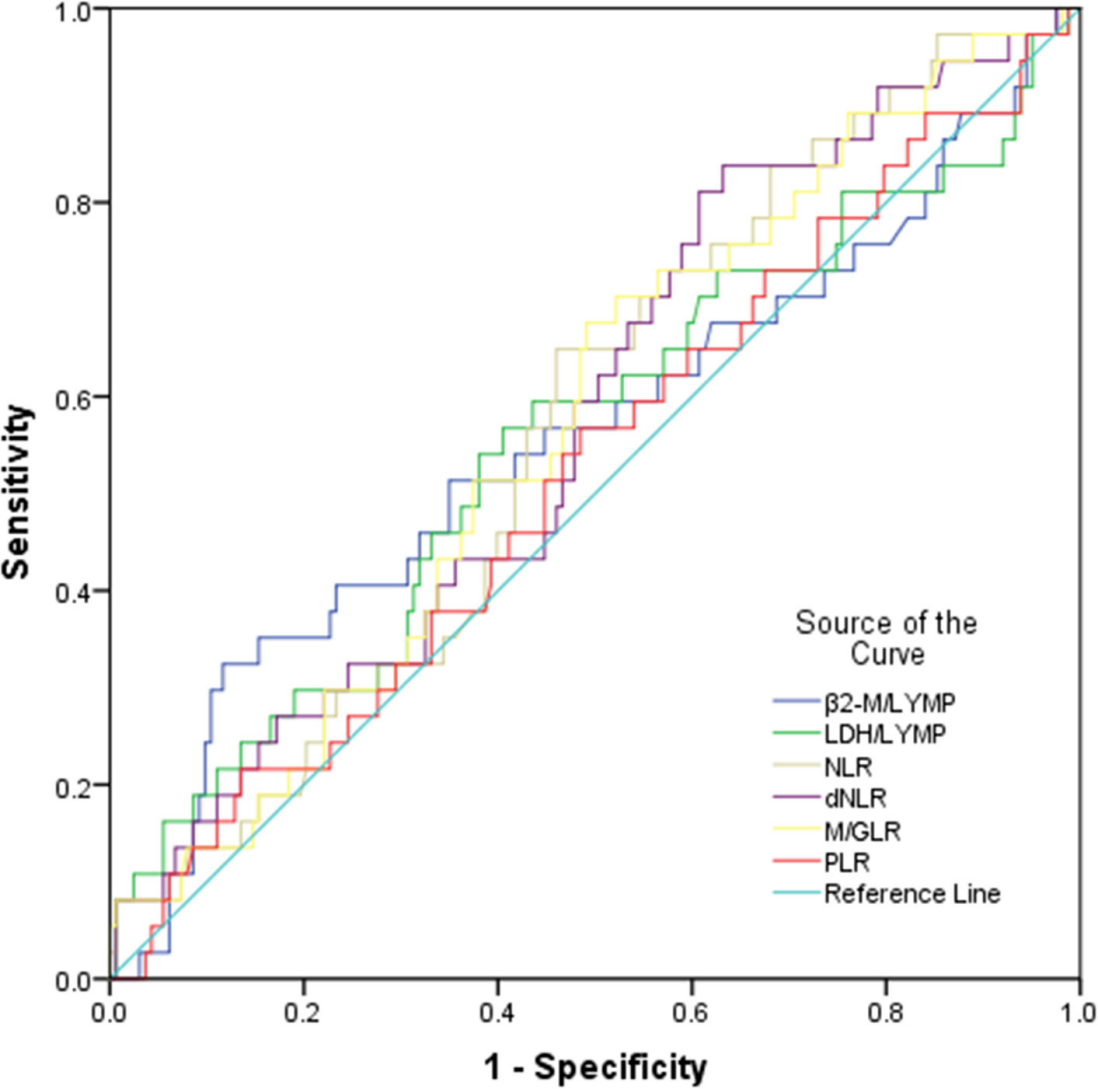
Assessment of cut-off value for βLRI, LLRI, NLR, dNLR, M/GLR and PLR in ENKTL patients prior to treatment Receiver operating characteristic (ROC) analysis was performed to evaluate the prognostic value of pretreatment βLRI, LLRI, NLR, dNLR, M/GLR and PLR. The area under the ROC curve value was 0.558, 0.559, 0.562, 0.567, 0.563 and 0.512, respectively.

Using overall survival rate as an endpoint, βLRI, LLRI, NLR, dNLR, M/GLR and PLR based stratification was performed using receiver operating characteristic curve (ROC) analyses. The area under receiver operating curve (AUC) for βLRI, LLRI, NLR, dNLR, M/GLR, PLR were 0.558, 0.559, 0.562, 0.567, 0.563, 0.512, and the optimal cut-off value corresponding to the maximum joint sensitivity and specificity were 4.87, 128.44, 2.36, 1.42, 2.65, 220.13, respectively (Figure 1).

### Correlation of clinical and pathological variables with βLRI and LLRI scores in ENKTL patients

We further investigated the relationship between pretreatment βLRI & LLRI and the clinical variables of ENKTL patients. Our data indicated that both of them correlated with paranasal extension (*p* = 0.023, *p* = 0.003) and KPI > 1 (*p* = 0.010, *p* = 0.002) as shown in Table 2. Moreover, βLRI also correlated with IPI > 1 (*p* = 0.002) and recurrence (*p* = 0.014), while LLRI correlated with B symptoms (*p* < 0.001). However, we did not observe any significant correlation between pretreatment βLRI & LLRI and other clinical or pathological parameters such as age, gender, Ann Arbor Stage, ECOG, and lymph nodes infiltration (all *p >* 0.05), as shown in Table 2.

**Table 2:** Correlation of βLRI and LLRI scores with clinical and pathological variables

Variables	Cases	βLRI		X^2^	*P* value	Cases	LLRI		X^2^	*P* value
		≤ 4.87	> 4.87				≤ 128.44	> 128.44		
**Age (years)**										
≤ 45	122	107 (87.7%)	15 (12.3%)	1.904	0.222	126	76 (60.3%)	50 (39.7%)	1.819	0.191
> 45	77	62 (80.5%)	15 (19.5%)			77	39 (50.6%)	38 (49.4%)		
**Gender**										
Male	140	117 (83.6%)	23 (16.4%)	0.675	0.517	144	82 (56.9%)	62 (43.1%)	0.017	1
Female	59	52 (88.1%)	7 (11.9%)			59	33 (55.9%)	26 (44.1%)		
**B symptoms**										
Yes	97	81 (83.5%)	16 (16.5%)	0.298	0.693	99	73 (70.2%)	31 (29.8%)	15.93	**< 0.001**
No	102	88 (86.3%)	14 (13.7%)			104	42 (42.4%)	57 (57.6%)		
**Ann Arbor Stage**										
IE	147	128 (87.1%)	19 (12.9%)	2.032	0.117	151	88 (58.3%)	63 (41.7%)	0.636	0.517
IIE	52	41 (78.8%)	11 (21.2%)			52	27 (51.9%)	25 (48.1%)		
ECOG										
0–1	191	164 (85.9%)	27 (14.1%)	3.274	0.102	195	112 (57.4%)	83 (42.6%)	1.244	0.297
> 1	8	5 (62.5%)	3 (37.5%)			8	3 (37.5%)	5 (62.5%)		
**Paranasal extension**										
Yes	86	70 (81.4%)	16 (18.6%)	5.940	**0.023**	88	42 (47.7%)	46 (52.3%)	9.657	**0.003**
No	61	58 (95.1%)	3 (4.9%)			63	46 (73.0%)	17 (27.0%)		
**Lymph Node Infiltration**										
Yes	45	36 (80%)	9 (20%)	1.102	0.343	45	91 (57.6%)	67 (42.4%)	0.259	0.614
No	154	133 (86.4%)	21 (13.6%)			158	24 (53.3%)	21 (46.7%)		
**LDH Elevated**										
Yes	55	43 (78.2%)	12 (21.8%)	2.699	0.121					
No	144	126 (87.5%)	1812.5%)							
**IPI**										
0–1	184	161 (87.5%)	23 (12.5%)	12.65	**0.002**	188	110 (58.5%)	78 (41.5%)	3.586	0.101
> 1	15	8 (53.3%)	7 (46.7%)			15	5 (33.3%)	10 (66.7%)		
**KPI**										
0–1	140	125 (89.3%)	15 (10.7%)	7.015	**0.010**	144	92 (63.9%)	52 (36.1%)	10.571	**0.002**
> 1	59	44 (74.6%)	15 (25.4%)			59	23 (39.0%)	36 (61.0%)		
**Recurrence**										
0	145	129 (89%)	16 (11%)	6.815	0.014	148	88 (59.5%)	60 (40.5%)	1.755	0.205
1	54	40 (74.1%)	14 (25.9%)			55	27 (49.1%)	28 (50.9%)		

### Independent prognostic factors in ENKTL patients

In addition, we also tried to identify any correlation of βLRI and LLRI with other clinical risk factors, PFS and OS by univariate analysis and Cox regression modeling. Our results revealed that pretreatment βLRI > 4.87 (*p* = 0.018), Ann Arbor stage > 1 (*p* = 0.022) and patients only with radiotherapy (*p* = 0.031) correlated with poor PFS of ENKTL patients. However, pretreatment βLRI > 4.87 (*p* = 0.023), LLRI >128.44 (*p* = 0.040), dNLR > 1.42 (*p* = 0.025), M/GLR >2.65 (*p* = 0.030) and Ann Arbor Stage > 1 (*p* = 0.008), ECOG > 1 (*p* = 0.009) along with IPI > 1 (*p* = 0.023) were identified to be significant predictors of poor OS in ENKTL patients, as shown in Table 3. Moreover, multivariate analysis demonstrated that Ann Arbor Stage > 1, βLRI > 4.87 and LLRI > 128.44 were significant independent predictors of poor OS, while Ann Arbor Stage > 1, βLRI > 4.87 were significant independent predictors of poor PFS (all *P* < 0.05), as shown in Table 4.

**Table 3:** Univariate analysis based identification of prognostic factors for PFS and OS in ENKTL patients

Variables	Progression-free survival			Overall survival		
	HR	95% CI	*P* value	HR	95% CI	*P* value
Age (≤ 45 or > 45)	1.149	0.675–1.958	0.609	1.535	0.805–2.926	0.193
Gender (Male or Female)	0.682	0.360–1.293	0.241	0.700	0.319–1.533	0.372
Ann Arbor Stage (IE or IIE)	1.940	1.102–3.416	**0.022**	2.482	1.268–4.859	**0.008**
LDH Elevated	1.203	0.673–2.151	0.532	1.410	0.707–2.813	0.329
ECOG (0–1 or > 1)	2.487	0.898–6.893	0.080	4.037	1.423–11.450	**0.009**
IPI (0–1 or > 1)	1.357	0.540–3.410	0.516	2.784	1.152–6.729	**0.023**
KPI (0–1 or > 1)	1.233	0.697–2.182	0.472	1.716	0.882–3.328	0.112
B symptoms (Yes or No)	1.354	0.802–2.303	0.254	1.336	0.696–2.565	0.383
βLRI (≤ 4.87 or > 4.87)	2.093	1.138–3.850	**0.018**	2.798	1.405–5.572	**0.003**
LLRI (≤ 128.44 or > 128.44)	1.549	0.911–2.626	0.104	1.977	1.030–3.794	**0.040**
NLR (≤ 2.36 or > 2.36)	1.045	0.618–1.767	0.870	1.479	0.771–2.838	0.239
dNLR (≤ 1.42 or > 1.42)	1.373	0.760–2.481	0.294	2.714	1.132–6.500	**0.025**
M/GLR (≤ 2.65 or > 2.65)	1.371	0.807–2.330	0.244	2.141	1.074–4.266	**0.030**
PLR (≤ 220.13 or > 220.13)	1.546	0.798–2.994	0.197	1.786	0.815–3.918	0.148

**Table 4:** Multivariate analysis based identification of prognostic factors for PFS and OS in ENKTL patients

Variables	Progression-free survival			Overall survival		
	HR	95% CI	*P* value	HR	95% CI	*P* value
Ann Arbor Stage (IE or IIE)	2.412	1.298–4.482	**0.005**	2.145	1.088–4.227	**0.028**
βLRI	2.888	1.445–5.770	**0.003**	4.409	1.973–9.856	**< 0.001**
ECOG (0–1 or > 1)				2.739	0.828–9.055	0.099
IPI (0–1 or > 1)				1.089	0.389–3.049	0.871
LLRI				2.864	1.345–6.097	**0.006**

### Development of a novel prognostic model

**Figure 2:**
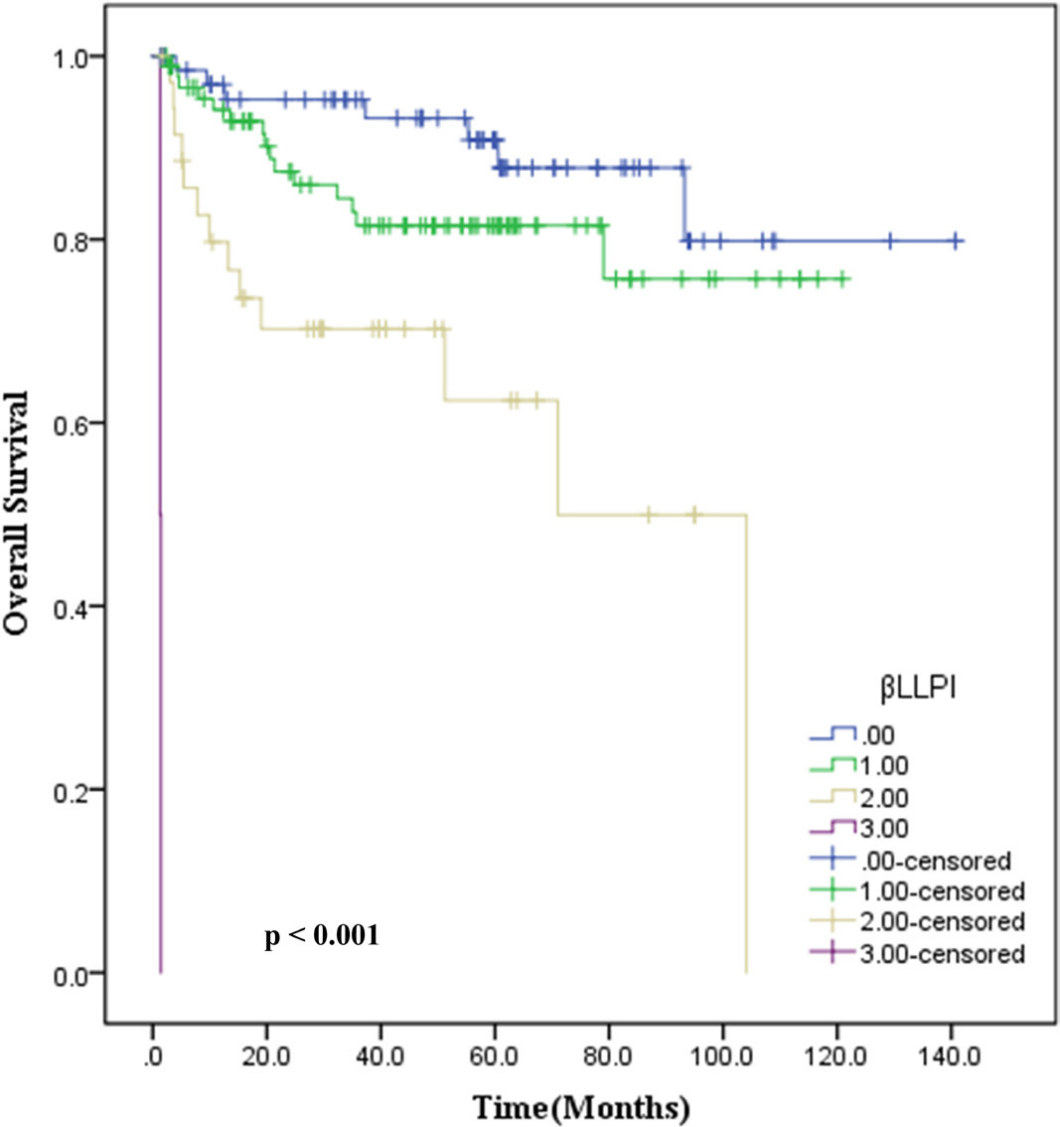
Estimation of overall survival with newly developed prognostic index (βLLPI) in patients with stage IE/IIE ENKTL, nasal type

Further we also sought to develop a novel prognostic model, based on the data from measurement of three variables (Ann Arbor stage, βLRI, and LLRI) in a cohort of 199 patients. The following criterion was used to develop this model: a score 0 indicated no adverse factors, while a score of 1, 2 or 3 represented one, two or three adverse factors, respectively. Based on this model, we observed that score 0 corresponded with 90% OS, whereas a score of 1, 2 or 3 indicated 83.5%, 63.9% and 0% OS, respectively. This novel prognostic model revealed the ability to discriminate outcomes between four groups of ENKTL patients (*p* < 0.001), as shown in Figure 2.

**Figure 3:**
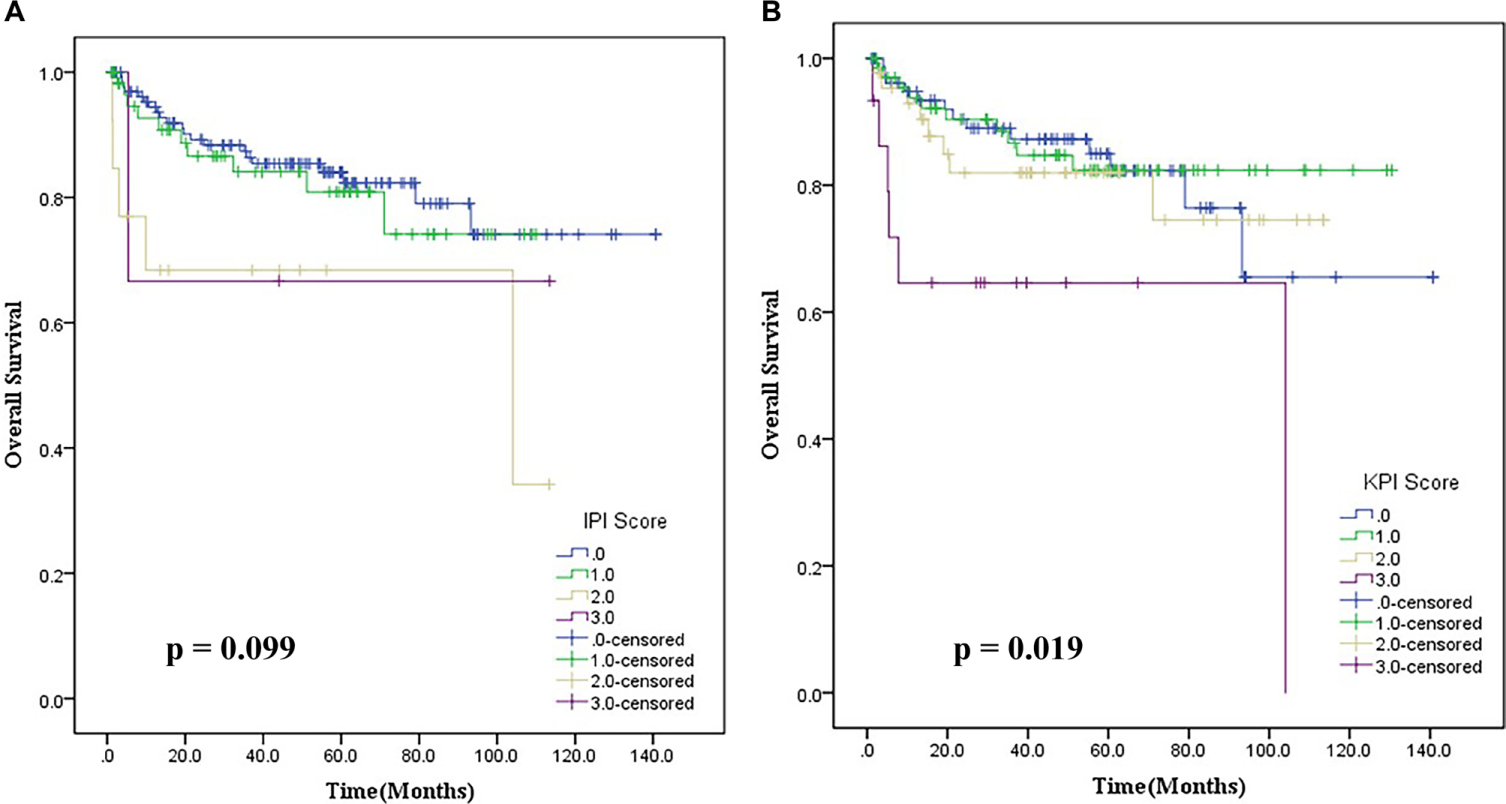
(**A**) Overall survival based on the International Prognostic Index (IPI) for patients with stage IE/IIE extranodal natural killer/T cell lymphoma, nasal type; (**B**) Overall survival based on the Korean Prognostic Index (KPI) for patients with stage IE/IIE extranodal natural killer/T cell lymphoma, nasal type.

Instead, the parallel comparison of ENKTL patient based on IPI and KPI prognostic models demonstrated that these models were not efficient in clearly discriminating patient outcomes. The IPI prognostic model classified 92.4% of patients into a low-risk group (0–1) and could not discriminate outcomes between all four groups (*p* = 0.099, Figure 3A). Similarly, the KPI prognostic model also classified patients as follows: 39.3% of the cases with score 0, 42.2% with score 1, 20.9% with score 2 and 7.1% with score 3, and was unable to discriminate outcomes between groups with a score 0 and 1 (*p* = 0.019), as shown in Figure 3B.

## DISCUSSION

Herein, we have assessed the prognostic value of βLRI and LLRI with other clinical factors in early-stage ENKTL patients. Our results indicated that βLRI and LLRI could be utilized in combination with Ann Arbor staging to predict the survival of ENKTL patients. To our knowledge, this is the first study to directly investigate the prognostic value of βLRI and LLRI in ENKTL.

Based on the assumption that high LDH, β2-MG and low lymphocyte counts may be associated with shorter survival in patients [10, 19–21], we studied the utility of βLRI and LLRI as a panel of prognostic biomarkers for ENKTL patients. Two recent studies have indicated that elevated aspartate aminotransferase (AST) to lymphocyte ratio index (ALRI) and AST to platelet ratio index (APRI) were associated with a poor prognosis in hepatocellular carcinoma patients [22–23]. However, there were no studies evaluating the prognostic value of βLRI and LLRI in patients with ENKTL, and thus, we focused on analyzing the role of pretreatment βLRI and LLRI in ENKTL prognosis.

We first identified the cut-off value of the inflammation-based prognostic scores according to ROC curve analysis, and a score of 4.87 and 128.44 appeared to be the optimal cut-off value for βLRI and LLRI with a maximum joint sensitivity and specificity. Although ROC-based cut-off optimization for dNLR and M/GLR enabled the stratification of ENKTL patients into high and low risk groups by univariate analysis, βLRI-based stratification was more optimal. Notably, βLRI cut-off determined by our analysis defined a relatively small subset of patients (15%) as high risk, and this subset of patients was associated with poor outcome. Furthermore, βLRI and LLRI retained their prognostic value on OS in multivariate analysis (*p <* 0.001 and *p* = 0.006 respectively). These observations led us to design a novel inflammatory marker-based prognostic model with three adverse factors, Ann Arbor Stage, βLRI and LLRI. The novel prognostic model was able to identify four categories of patients with significantly different prognoses (*p* < 0.001).

To further validate the valuable clinical practice of our model, we compared the new model with existing systems. The IPI failed to distinguish the outcomes of ENKTL patients, which may be partly accounted for the uneven distribution of patients within risk groups and inefficiency to distinguish between low-risk and low-intermediate-risk groups [24]. Compared to IPI, the KPI displayed a homogenous patient distribution, and had the ability to discriminate low and high-risk groups. However, as reported previously, it failed to separate patients in low-risk group [7]. Thus neither IPI nor KPI were suitable to predict prognosis for early stage ENKTL patients, as the majority of these patients were categorized as low or low-intermediate risk groups. However, our prognostic model displayed superior predictive ability for these patients.

In conclusion, our study clearly established that pretreatment βLRI and LLRI seems to be independent prognostic factor candidates for ENKTL patients, and our novel βLRI and LLRI based model had the ability to stratify patients into four groups with a higher prognostic discrimination, in comparison to IPI or KPI. However, future prospective studies are required to further validate these results.

## MATERIALS AND METHODS

### Study population

We recruited 211 previously untreated ENKTL patients, who were histologically diagnosed according to 2008 World Health Organization classification, and staged IE/IIE, according to Ann Arbor system in Cancer Hospital of Academy of Medical Sciences (CAMS) & Peking Union Medical College (PUMC) between January 2003 and December 2015. The patients’ characteristics including age, gender, ECOG score, B symptoms, white blood cell, lymphocytes, monocyte, neutrophil, platelet and levels of serum lactate dehydrogenase (LDH) and beta2-microglobin (β2-MG), were collected for analysis. In addition, computed tomography (CT) and magnetic resonance imaging (MRI) of the head and neck, CT of the chest, abdomen, & pelvis, along with bone marrow examination or positron emission tomography/computed tomography (PET/CT) scans, were used for clinical staging. The paranasal stage IE was defined as the lesion extending to adjacent tissues or organs. However, limited stage IE was defined as tumors confined to the nasal cavity [25]. In addition, IPI and KPI were also assessed.

### Treatment and follow-up

The following treatment regimens were used in all patients. Among the total patients, 114 patients received combined chemotherapy and radiotherapy, while the other 97 patients only received radiotherapy. Chemotherapy regimens included CHOP (cyclophosphamide, doxorubicin, vincristine and prednisone), CHOPE (cyclophosphamide, doxorubicin, vincristine, prednisone, and etoposide), GDP (gemcitabine, dexamethasone, and cisplatin), and IMVP-16 (ifosfamide, etoposide, methotrexate). The median follow-up time for all 211 patients was 47.8 months (range, 1.1 to 140.7 months). β2-MG was not assessed in 12 patients, thus only 199 patients with this information were analyzed.

### Statistical methods

Receiver operating curve (ROC) analysis was used to define the optimal cut-off value for βLRI and LLRI scores. The associations of these scores with clinical and pathological parameters were estimated by using Chi-square test or Fisher's exact test. Overall survival (OS) was calculated from the date of treatment to date of death caused due to any reason or until the last follow-up period. Similarly, progression free survival (PFS) was assessed from the date of diagnosis to first progression or recurrence after initial response or last follow-up or death. The Kaplan–Meier method along with the log-rank test was used to calculate survival curves. Univariate analysis was used to evaluate the prognostic factors for OS, and factors with a *P value* of < 0.05 were further analyzed using multivariate analyses by Cox proportional hazards model. All tests were two sided, and *p* < 0.05 represented statistical significance. All data was analyzed using SPSS version 23.0 (SPSS Inc, Chicago, IL) software.

## Abbreviations

βLRI: β2-microglobin (β2-MG) to lymphocytes ratio index score
LLRI: lymphocytes ratio index score
ENKTL: extranodal natural killer/T cell lymphoma
NLR: neutrophil-lymphocyte ratio index
dNLR: derived neutrophil-lymphocyte ratio index
M/GLR: monocyte/granulocyte to lymphocyte ratio
PLR: platelet-lymphocyte ratio index
